# Rapid Detection of Food Allergens by Microfluidics ELISA-Based Optical Sensor

**DOI:** 10.3390/bios6020024

**Published:** 2016-06-07

**Authors:** Xuan Weng, Gautam Gaur, Suresh Neethirajan

**Affiliations:** Bionano Lab, School of Engineering, University of Guelph, Guelph, ON N1G 2W1, Canada; xuanw@uoguelph.ca (X.W.); ggaur@uoguelph.ca (G.G.)

**Keywords:** food allergen, gluten, Ara h 1, microfluidics, optical sensor

## Abstract

The risks associated with the presence of hidden allergens in food have increased the need for rapid, sensitive, and reliable methods for tracing food allergens in commodities. Conventional enzyme immunosorbent assay (ELISA) has usually been performed in a centralized lab, requiring considerable time and sample/reagent consumption and expensive detection instruments. In this study, a microfluidic ELISA platform combined with a custom-designed optical sensor was developed for the quantitative analysis of the proteins wheat gluten and Ara h 1. The developed microfluidic ELISA biosensor reduced the total assay time from hours (up to 3.5 h) to 15–20 min and decreased sample/reagent consumption to 5–10 μL, compared to a few hundred microliters in commercial ELISA kits, with superior sensitivity. The quantitative capability of the presented biosensor is a distinctive advantage over the commercially available rapid methods such as lateral flow devices (LFD) and dipstick tests. The developed microfluidic biosensor demonstrates the potential for sensitive and less-expensive on-site determination for rapidly detecting food allergens in a complex sample system.

## 1. Introduction

Food allergies have become an important food safety and health concern worldwide. In North America, 6% of young children and up to 4% of adults suffer from food allergies [1]. Virtually any food can cause an allergic reaction in a susceptible individual. Clinical symptoms of food allergies can range from minor digestive disorders and skin irritations to severe, potentially life-threatening symptoms. The main ways to manage food allergies are allergen avoidance or treatment of symptoms [2]. Nevertheless, the detection of food allergens in the food matrix can be a very hard task because food allergens are usually present only in trace amounts and because sensitized individuals are reliant upon food labels to identify the possible presence of an allergenic ingredient.

Wheat and peanut are considered the two of the eight most prevalent allergenic foods (milk, eggs, peanuts, tree nuts, fish, soy, wheat, and crustacean shellfish) in the United States. Gluten is a complex mixture of water-insoluble proteins (gliadin and glutenin) found in wheat and related grains, associated pathologies of which are food allergens, coeliac disease, and gluten sensitivity [3]. Food allergy associated with gluten intake affects 0.2%–0.5% of the population [3] and gluten sensitivity affects nearly 10% of the US population [4]; it is especially prevalent among people with coeliac disease (CD). For persons with gluten sensitivity, the only treatment is the adoption of a gluten-free diet. The U.S. Food and Drug Administration (FDA) has defined the term “gluten-free” to mean foods that contain less than 20 ppm gluten. Ara h 1 is one of the major peanut allergens present in peanut and other peanut products, including candies, cereals, and baked goods; peanut is also one of the main foods associated with severe, potentially fatal allergic reactions, including life-threatening anaphylaxis [5].

Peanut and tree nut allergies were reported to affect more than 1% of the US population and appear to be increasingly reported among children [6]. The allergenicity of peanut is heat-resistant because no reduction [7,8] or even an increase [9] of IgE binding to Ara h 1 protein occurs due to the structural conformation changes upon the heat treatment [10]. Consumers can be exposed to the risk of peanut allergens when foods become contaminated from shared production lines, raw materials, or ingredient supply chains [11]. Therefore, sensitive individuals with a peanut allergy should avoid products that bear precautionary statements on the label. However, the use of advisory labels is voluntary, and not all manufacturers do so. The risk associated with the presence of allergens in food has increased the need for rapid, accurate, cost-effective, and sensitive analytical methodologies for the detection of food allergens.

Currently, immunoanalytical methods are still the preferred methodologies being used to detect food allergens [12]. Among these, enzyme immunosorbent assay (ELISA) is the most popular and commonly used methodology for the routine monitoring of allergens due to its sensitivity, high precision, and good potential for standardization [13,14,15]. ELISA is also the dominant protocol for most commercial kits designed for quantitating low levels of food allergens in food ingredients as well as in prepared and processed foods and beverages [14]. However, conventional ELISA has usually been done in a centralized lab with standard laboratory equipment and a microtiter plate spectrophotometer, the fee for sample delivery and storage, testing is expensive and oftentimes leads to delays in acquiring results. Consequently, there is an ongoing demand for analytical strategies that can be used outside the laboratory environment to assess the safety and quality of foods on-site. Although some simple, rapid on-site assays have become commercially available (Reveal 3-D™ Test; Crystal Chem^®^ Lateral Flow Kit; Agrastrip^®^ Allergen Test Strips), they are only able to provide qualitative results. More recently, biosensors have been developed that appear to be as powerful, sensitive, selective, and, in some cases, capable of real-time measurement, effectively replacing traditional methodologies such as mass spectrometry and immunological assays [2].

A biosensor refers to an integrated bioreceptor-transducer device capable of converting a biorecognition event into a measurable signal that depends on the concentration of the target analyte. Optical biosensors based upon absorption, fluorescence, and surface-plasmon resonance (SPR) are the most widely studied for food allergen analysis [16]. SPR is effective because it provides real-time results, is automated, and has high surface sensitivity. SPR biosensor technology is based on changes in the refractive index at the surface of a sensor chip, which is caused by binding between the specific antibodies immobilized on the chip surface and the allergens in the sample. Though the SPR biosensors have the advantages of reducing assay time and providing a high degree of automation, the majority of these systems are relatively expensive due to their use of high-performance optical machines and chips [17]. It is expected that simple, rapid, low-cost, and reliable biosensors with high sensitivity for food allergen detection will become available and will come to be applied in food control agencies.

Microfluidics has become an increasingly popular and powerful tool in biochemistry applications [18] and presents distinctive advantages, such as faster reaction times, lower sample consumption, greater sensitivity, small dimensions, short diffusion distance, and high surface tension [19,20]. Many studies have described immunoassays in microfluidic devices employing various detection methods such as fluorometric and colorimetric measurement and SPR, but few have been applied to the detection of food allergens [21,22,23]. However, most of the strategies using only microfluidic chips require bulky and expensive instruments or the use of complex chip fabrication processes, which limit their application in making portable devices.

The integration of microfluidics technology in biosensors will help contribute to the development of low-cost, fully integrated point-of-care (POC) diagnostics in addition to the distinct advantage of substantially reducing sample/reagent consumption and assay time. In keeping with the theme of low-cost diagnostics, the objective of this study was to demonstrate the integration of the microfluidic ELISA chip with a custom-designed optical sensing device for rapid detection of food allergens.

## 2. Materials and Methods

### 2.1. Reagents and Materials

The Gluten (Gliadin) ELISA Kit and the Ara h 1 ELISA kit were purchased from Crystal Chem Inc. (Downers Grove, IL, USA) and INDOOR Biotechnologies Inc. (Charlottesville, VA, USA), respectively. Anti-gliadin antibody 14D5, HRP-conjugated rabbit anti-gliadin polyclonal antibody, and 3, 3′, 5, 5′-Tetramethylbenzidine (TMB) substrate were obtained from Abcam Inc. (Toronto, ON, Canada). Monoclonal antibody 2C12 (clone 2C12 A11 A3) and biotinylated antibody 2F7 (clone 2F7 C12 D10) to be used in on-chip ELISA were obtained from the Ara h 1 ELISA kit. Polydimethylsiloxane (PDMS, Sylgard 184) was purchased from Dow Corning (Midland, MI, USA). All other reagents and chemicals were purchased from Sigma-Aldrich Co. (Oakville, ON, Canada). Food containing peanut and gluten products, biscuits, and gluten-free flour were obtained from a local grocery store in Guelph, Canada.

### 2.2. Preparation of Food Samples

Food samples for gluten detection were firstly ground to homogeneity, and 0.5 g of the homogenized sample was mixed with 9.5 mL of the sample extraction solution provided by the kit. The mixture was capped tightly, vortexed for 30 s, and then put in the boiling water bath for 10 min. After being cooled down under running water, the tube was vortexed for 30 s. The solution mixture was centrifuged at 3000× *g* for 20 min at room temperature. The supernatant of the mixture was filtered through a filter syringe (GHP Membrane Disc Filters, VWR International; Suwanee, GA, USA) with 0.2 μm diameter pores. The filtrate was used to make a series of dilutions, which would be used as the working sample solutions for microfluidic ELISA. Food samples for Ara h 1 detection followed the protocol of the gluten assay.

### 2.3. Design and Fabrication of Microfluidic Chip

**Figure 1:**
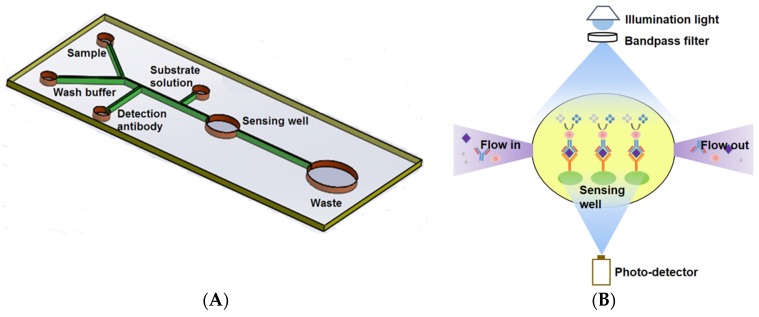
Schematic of the Microfluidic ELISA chip layout (not to scale) (**A**) and Principle of the on-chip absorption detection with the miniaturized optical sensor device (**B**). The diameters of the sensing and waste wells are 2.5 mm and 6 mm, respectively. The width of the main channel and the branch channels are 200 μm and 100 μm, respectively. All other wells are 0.75 mm in diameter to fit the loading syringe tip.

The microfluidic chip consisted of four inlets and one outlet. The inlets with associated channels were used to load the samples, the enzyme-conjugated antibody, and the substrate solution as shown in Figure 1A. Solutions were sequentially dispensed by hand. The fabrication of the microfluidic chip followed standard photolithography and soft lithography [24]. Briefly, it involved making a silicon wafer master mold and Polydimethylsiloxane (PDMS) chips. The master mold bearing the desired microchannel layout was made by depositing a layer of 80 μm UV cured SU-8 2025 negative photoresist (MicroCHEM, Westborough, MA, USA) onto the surface of the wafer. SU-8 photoresist was firstly deposited dynamically onto the silicon wafer with a spin-coating speed at 500 rpm for 25 s followed by 1000 rpm for 30 s to obtain the desired thickness. After soft baking at 65 °C for 1 min, 95 °C for 9 min and cooling down, the photoresist coated wafer was covered by a photomask bearing desired microchannel geometry and exposed to UV light of a total energy of 215 mJ/cm^2^. A post-exposure bake was then conducted at 65 °C for 2 min, 95 °C for 7 min. After cooling down, the wafer was place into SU-8 developer until the unexposed photoresist was completely rinsed off. A master was obtained after hard bake for 30 min to strengthen the bonding. A PDMS chip was made with a soft lithography technique by using the master mold. A degassed mixture of PDMS prepolymer and curing agent (10:1 *w/w*, Sylgard, Dow Corning, Burlington, ON, Canada) was poured onto the mold and cured for 4 h at 75 °C. The solidified PDMS replica was then peeled off of the master mold, punched (Harris Uni-coreTM, Ted Pella Inc., Redding, CA, USA) to form the inlets and outlet, and bonded onto a glass slide (25 mm × 75 mm × 1 mm, VWR International, Suwanee, GA, USA) after oxygen plasma treatment for 40 s. A position marker was used to facilitate the alignment between the sensing well and the sensing window of the photo-detector mounted in a custom-built container (10 × 6 × 5 cm^3^) when conducting the bonding. A schematic of the custom-built container for the optical and electrical components assembly and alignment can be found in previous work [25].

### 2.4. Antibodies Immobilization

The optimization of antibody immobilization on the PDMS surface was performed by comparing the coating efficiency of two different methods, (1) passive adsorption (PA) and (2) functionalization with APTES, to generate amino groups and covalent immobilization (CI).

For passive adsorption, the capture antibodies were immobilized directly onto the sensing surface by hydrophobic interactions [24,26]. The PDMS and glass slide underwent plasma treatment for 30 s to form hydrophilic silanol (Si-OH) for binding. To make the microchannel wall fully recover to the hydrophobicity, the microchannel was exposed to air overnight before antibody immobilization.

For covalent immobilization, before the immobilization of the capture antibody, the PDMS/glass surfaces functionalization was firstly performed in accordance with the protocol reported by Yu *et al.* [27]. A mixture of deionized (DI) water, hydrogen chloride (HCl), and hydrogen peroxide (H_2_O_2_) in a ratio of 5:1:1 was made as the oxidative solution to oxidize the surface. After rinsing the microchannel with DI water and ethanol, (3-aminopropyl)triethoxylsilane (APTES)/ethanol (1/1, *v/v*) was dispensed into the microchannel and incubated for 30 min. The microchannel was rinsed again with ethanol and DI water to remove the excess APTES. After the above oxidation and pretreatment steps, a NaIO_4_/dextran solution was dispensed into the microchannel for 1 h and flushed with DI water. Finally, 0.1 mol/L NaIO_4_ was pumped into the microchannel and incubated for 1 h. After washing with DI water, the microfluidic chip was ready for antibody immobilization.

The capture antibody was then immobilized on both the hydrophobicity-recovered and APTES-functionalized microchannels. The capture antibody, diluted with the coating buffer at a concentration of 10 μg/mL, was dispensed into the microchannel and incubated for 2 h at room temperature to allow for binding. The microchannel was then rinsed with PBS to remove the unbound antibody and was followed by dispensing BSA in the PBS buffer into the microchannel and incubating it for 15 min at room temperature to block the unreacted surface in order to reduce non-specific binding. The microchannel was then rinsed with PBS, and the microfluidic chip coated with primary antibodies was ready to use.

### 2.5. On-Chip ELISA Test Procedure

Firstly, 5 μL of standard or sample extraction solution was gently dispensed into the microchannel and incubated for 10 min to allow the antigen present in the solution to be captured on the surface by the immobilized capture antibody. Secondly, the microchannel was washed with a wash buffer twice. The HRP-labelled detection antibody was then dispensed into the microchannel. After 5 min of incubation, the microchannel was rinsed with the wash buffer twice again and followed with the TMB substrate. After the substrate was added, readings were taken at 605 nm by the custom-design optical sensor at intervals of 1, 3, and 5 min. For the peanut allergen (Ara h 1) assay, after washing to remove the unbound antigen, diluted biotinylated antibody specific to Ara h 1 was dispensed into the microchannel and was then rinsed twice. Peroxidase conjugated streptavidin was then added and incubated for 5 min. Lastly, the microchannel was washed, and the 1 mmol/L ABTS in 70 mmol/L citrate phosphate buffer was added. After the addition of substrate, the light intensity was read at 405 nm at time intervals of 1, 3, 5, and 7 min.

To compare the performance of the microfluidics-based optical sensor with the commercial kit, the same gluten working solutions and sample solutions were measured with both the presented biosensor and the commercial kits. Detailed procedures for commercial kits can be found elsewhere [28,29]. Matrix spikes were prepared by adding standard solutions of known concentrations into working sample solutions followed by vortex at final spiked concentrations of 20, 40 μg/g (gluten sample) and 50, 100 μg/g (Ara h 1 sample), respectively. The precision of this method in terms of recovery rate and the RSD were evaluated by adding standard solutions into the same group of samples.

### 2.6. Optical Sensor

To make the system cost-effective and potentially useful as a handheld device, a simple, miniaturized, and sensitive optical sensor was developed based on the light absorption measurement. The main components of the sensor were an LED (405 nm for Ara h 1, LUXEON UV, Digi-Key; 617 nm for gluten, Luxeon Rebel, Luxeon Star LEDs, Brantford, ON, Canada) with a single-band bandpass filter (405/10 nm for Ara h 1, 600/37 nm for gluten, Semrock, Rochester, NY, USA) providing illumination and a high sensitivity photo-detector (S8745-01, Hamamatsu, Bridgewater, NJ, USA) that converts light intensity to an electrical signal for reading. In an ELISA assay, the biorecognition event occurred in the sensing well, and the light absorption of the resultant complex was proportional to the concentration of the target analyte (antibody). Therefore, the transmitted light intensity through the sensing well was measured by the photo-detector and was used to evaluate the concentration of samples. A schematic of the optical sensor is shown in Figure 1B.

## 3. Results

### Determination of Optimal Immobilization Strategy

**Figure 2:**
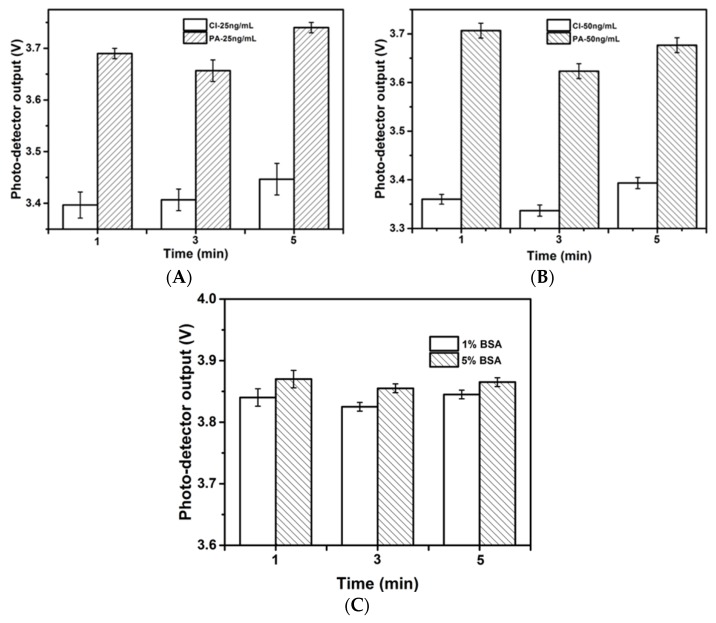
Photo-detector biosensor output of optical absorption detection upon gluten standard solution of 25 ng/mL (**A**) and 50 ng/mL; (**B**) by two different antibody immobilization strategies, passive adsorption (PA) and covalent immobilization (CI). Optimization of the blocking step by using blocking buffer of 1% (w/v) and 5% (w/v) BSA in PBS for gluten standard solution of 50 ng/mL; (**C**) Experiments were conducted without coating antibodies on the microchannels. The higher concentration of the blocking buffer improved the blocking efficiency.

A series of standard solutions were compared in the on-chip ELISA assay on the antibody-immobilized microchannel by two different methods, *i.e.*, (1) passive adsorption (PA); and (2) covalent immobilization (CI). It is known that the virgin PDMS surface is hydrophobic and is good for the passive adsorption of protein molecules; however, it has also been shown that this hydrophobicity is greatly reduced by 30 s of plasma oxidation [24]. Plasma oxidation was conducted on PDMS to introduce hydrophilic silanol groups for microchannel formation (binding). The chips were therefore left overnight after the completion of this step to allow the hydrophobicity of the channel wall to fully recover. The protocol for CI was drawn from an established protocol [27]. As shown in Figure 2A,B, for samples at the same concentration, the output of the photo-detector after PA was higher than that after CI, indicating a weaker light absorption capacity of the resultant complex. This difference may be attributed to lower antibody immobilization efficiency because the passive adsorption of antibodies can cause protein denaturation and can reduce protein functional sites or activities [30,31]. Therefore, the CI method was applied in the subsequent experiments. The repeatability and reproducibility were evaluated by the relative standard deviations given in the figure, and the CI method showed better repeatability with RSD of 3.0% less than the PA method. Non-specific binding, which results from a decrease of detection sensitivity due to the avidity of the PDMS walls, is another concern for the microchannel surface. To resolve this problem and to reduce background interference, the use of a blocking step is very important. In this study, the antibody-immobilized channel surface was blocked with BSA blocking buffer to saturate excess protein-binding sites on the channel wall. To optimize BSA blocking, two concentrations of BSA blocking buffer (1% w/v and 5% w/v) were compared. Experiments were conducted without coating antibodies to investigate the nonspecific binding effect, in which the signals were generated by the nonspecific binding of antibody on the PDMS walls. As shown in Figure 2C, a higher concentration of the BSA blocking buffer provided an improved blocking efficiency. This result may be attributed to the larger surface to volume ratio of the microchannel, which provided considerably more binding sites. Hence, nonspecific absorption may occur when the blocking buffer is insufficient to cover all the binding sites on the channel wall. In all the microfluidic ELISA assays based experiments, 5% w/v of BSA blocking buffer was applied.

## 4. Gluten and Ara h 1 Assay

**Figure 3:**
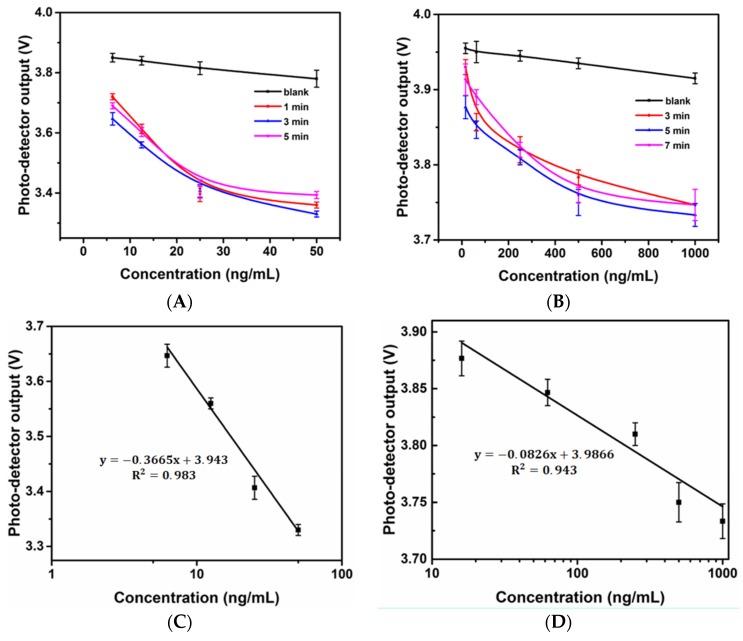
Time-dependent response of the developed optical microfluidic biosensor upon sensing the gluten standard solution (**A**) and Ara h 1 standard solution (**B**); Linear standard curves of gluten (**C**) and Ara h 1 (**D**).

The incubation time of each individual step of the microfluidic ELISA biosensor decreased in proportion to the volumes. However, relatively long times were still needed to complete antibody-allergen binding to achieve maximum sensitivity. In addition, the time-dependence of the enzyme reaction was investigated because the light intensity captured by the photo-detector was affected by the incubation time. Figure 3A shows the response of the optical sensor to wheat (gluten) standard solutions with different reaction times. As seen in the figure, 3 min can be considered to be the saturation time based on the enzyme kinetics for the enzyme reaction under the associated volume because the outputs of 1 min and 5 min were higher than those of 3 min. Similarly, the response of the optical sensor to the Ara h 1 standard solution with different reaction times is shown in Figure 2B. Five min was considered to be the saturation time. The 3-min and 5-min curves served as the standard curves for gluten and Ara h 1 with the custom-made biosensor, respectively. As shown in the Figure 3C,D, an R^2^ value of 0.983 was found in linear response region between 6.25 ng/mL and 50 ng/mL for gluten and 0.943 in linear response region between 16 ng/mL and 1000 ng/mL for Ara h 1. The calculated detection limits [32] of the gluten and Ara h 1 assays were 4.77 ng/mL and 15.2 ng/mL, respectively.

Gluten concentrations in regular biscuits and gluten-free flour and Ara h 1 concentrations in chocolate and peanut butter were assayed by the microfluidic ELISA chip. The same samples were simultaneously measured with commercial kits and read by a microplate reader for comparison. Actual concentration was calculated by multiplying the reading from the standard curve with the dilution factors. The results are given in Table 1. The precision of our method was examined by a spiking experiment, followed by calculation of the recovery rate and its relative standard deviation (RSD). The recovery rates were acceptable, in the range of 89.8%–107.9% as shown in Table 2, and the repeatability was good with RSD less than 3.1%.

The Ara h 1 assay was not as sensitive as the gluten assay and required more time to reach a stable readout, which may be due to the result of the food sample preparation. For example, roasted peanut was avoided because it has been shown to decrease protein extraction efficiency [33]. Food preparation processing risks altering the integrity of protein and DNA [34] from the samples, results in the decreased sensitivity of detection. Hence, in our study, food sample preparation for the Ara h 1 assay did not use heating. Sample preparation from foods is therefore particularly important when quantifying the allergen content of food products and will need to be further studied to improve the sensitivity of the microfluidic ELISA method in Ara h 1 assays.

## 5. Conclusions

We have demonstrated that the developed microfluidic biosensor significantly reduces the detection time from 4 h to 20 min and also reduces the use of costly reagents and samples (up to 20 folds) while maintaining superior sensitivity, and compares favorably to commercial ELISA kits for wheat gluten and Ara h 1 in food samples. In conclusion, a low-cost, rapid, miniaturized, and highly sensitive microfluidic ELISA device for the detection of food allergens was developed. The developed microfluidic biosensor has the potential to provide food safety inspectors and industries with the flexibility to select assay protocols for specifically targeted allergens, making it an extremely versatile platform for ELISA applications. We believe that our developed method is ideally suited to become a sensitive and less expensive on-site determination for rapidly detecting food allergens in a complex sample system. Further optimization of the sensing system to enhance the antibody immobilization efficiency and sensitivity is warranted.

## Figures and Tables

**Table 1:** Comparison of gluten/Ara h 1 detection of food samples by commercial kits and the developed microfluidic ELISA biosensor.

Allergen	Food Sample	Concentration Mean (ppm or μg/g) ± RSD (%)	
		Commercial Kit	On-Chip ELISA
Gluten	Biscuit	37,166 ± 10.8	36,948 ± 12.2
	Gluten free flour	4.93 ± 9.7	4.77 ± 14.6
Ara h 1	Chocolate bar	63.26 ± 16	55.4 ± 11.2
	Peanut butter	3043 ± 14.2	3232 ± 19.1

**Table 2:** Determination of gluten/Ara h 1 in spiked samples.

Sample	Spiked (ppm or μg/g)	Measured by Biosensor (ppm or μg/g)	Recovery by Biosensor (%)	RSD (%)
Gluten free flour	0	5.0		
	20	23.0	89.8	3.1
	40	41.9	92.3	1.3
Chocolate bar	0	67.1		
	200	277.7	105.3	1.8
	400	498.9	107.9	1.1

## Abbreviations

The following abbreviations are used in this manuscript:

ELISA: Enzyme-linked immunosorbent assay; CD: celiac disease; FDA: Food and drug administration; SPR: Surface plasmon resonance; POC: Point-of-care; CI: Covalent immobilization; PA: Passive adsorption; BSA: Bovine serum albumin; PDMS: Polydimethylsiloxane; LFD: Lateral flow devices; RSD: Relative standard deviation; LED: Light-emitting diode; HRP: Horseradish peroxidase; PBS: Phosphate-buffered saline; HCL: hydrogen chloride; H_2_O_2_: hydrogen peroxide; DI: Deionized; ABTS: 2,2'-Azinobis [3-ethylbenzothiazoline-6-sulfonic acid]-diammonium salt; TMB: 3,3',5,5'-tetramethylbenzidine; APTES: (3-Aminopropyl)triethoxysilane.
